# Early Post-Operative Endoscopy Is Associated with Lower Surgical Recurrence of Crohn's Disease: A Retrospective Study of Three Successive Cohorts

**DOI:** 10.1155/2022/6341069

**Published:** 2022-11-01

**Authors:** Caroline Amicone, Carla Coimbra Marques, Catherine Reenaers, Catherine Van Kemseke, Laurence Seidel, Edouard Louis

**Affiliations:** ^1^Departement of Gastroenterology, Centre Hospitalier Universitaire de Liège, Université de Liège, Liège, Belgium; ^2^Departement of Abdominal and Transplantation Surgery, Centre Hospitalier Universitaire de Liège, Liège, Belgium; ^3^Departement of Biotatistics, Centre Hospitalier Universitaire de Liège, Liège, Belgium

## Abstract

**Background:**

The severity of endoscopic recurrence during the first year after intestinal resection for Crohn's disease is predictive of clinical recurrence. The aim of our study was to assess the impact of the implementation of an ileocolonoscopy during the first year after surgery on surgical recurrence.

**Methods:**

All patients who underwent a first intestinal resection for Crohn's disease between 1992 and 2018 at the University Hospital of Liège were retrospectively included. The time to surgical recurrence was compared in three successive groups of patients operated on in the period 1992–2001 (group A), 2002–2011 (group B), and 2012–2020 (group C) using the Kaplan–Meier method and the Log-Rank test. To identify independent prognostic factors, a multivariate analysis was used via the Cox model.

**Results:**

223 patients (group A = 69, group B = 94, group C = 60) were included. Probabilities of surgical recurrence were significantly lower in group C (2.2% and 4.7% at 3 and 5 years, respectively) compared with group B (4.2% and 7.6% at 3 and 5 years, respectively) and with group A (9% and 18.2% at 3 and 5 years, respectively) (*p* = 0.0089). Ileocolonoscopy during the year after surgery was associated with a significantly reduced surgical recurrence rate in univariate and multivariate analysis (HR = 0.31, *p* = 0.0049).

**Conclusion:**

The implementation of an early ileocolonoscopy after surgery for Crohn's disease since early 2000 has been associated with a reduced surgical recurrence over the last 30 years.

## 1. Introduction

Crohn's disease (CD) is a chronic inflammatory bowel disease that can affect any part of the gastrointestinal tract with a preference for the distal small intestine and the proximal colon.

It is considered today as a progressive disease with multiple inflammatory flares that lead to cumulative tissue damage and complications such as fistulas and strictures requiring surgical resection [1, 2]. Despite recent advances in therapeutic management, surgery is still required in a significant proportion of patients [3]. Almost half of the patients, 10 years after diagnosis, must undergo surgery [2] and a third will need multiple surgical resections during the course of their disease [4]. Resection is definitely not a curative solution and CD frequently reappears, most often near anastomosis. In fact, 70–90% of patients present with endoscopic lesions within 12 months following surgery and it has been shown that new Crohn's lesions develop in the neo-terminal ileum in the months or even weeks after surgery [5, 6]. These endoscopic lesions graded according to the Rutgeerts score are, most of the time, present without clinical symptoms, precede clinical relapse, and are located near the anastomosis [7]. The severity of the lesions discovered during early postoperative endoscopy appears to be predictive of the clinical and surgical recurrence of the disease [7, 8].

The European Crohn's and Colitis Organisation (ECCO) recommends since the beginning of the 2000s an endoscopy within 6 to 12 months after surgery with a Rutgeerts score and therapeutic adaptation according to the score [9, 10].

However, the level of evidence suggesting the impact of this strategy on clinical and surgical relapse remains low. A retrospective study, carried out in 132 patients from 1995 to 2005, compared the clinical relapse rates in patients who had a colonoscopy between 6 to 12 months postoperatively with adaptation of the treatment and those who did not have a postoperative endoscopy [11]. The rates of clinical recurrence were significantly lower in the group with colonoscopy (21% and 26% at 3 and 5 years, respectively) compared to the group without colonoscopy (31% and 52% at 3 and 5 years, respectively). More recently, the prospective randomized and controlled POCER trial demonstrated that a preventive treatment adapted to the risks of recurrence and a therapeutic adaptation according to the early endoscopic recurrence at 6 months, reduced the severity of endoscopic lesions at 18 months [12]. These are supportive data, but the most important and robust outcome is probably the surgical recurrence, reflecting disease progression and accumulating tissue damage.

The aim of this mono-centric retrospective study was to assess the evolution of surgical recurrence over the last three decades and the impact of early post-operative endoscopy on this outcome and to determine the factors associated with this surgical relapse.

## 2. Materials and Methods

### 2.1. Study Population and Design

We performed a single-center retrospective study. The selection of patients was carried out from the surgical database of patients with inflammatory bowel disease followed at the University Hospital CHU Liège, and who underwent one surgical resection in the course of their CD between January 1992 and September 2018. The data collection and the study protocol were accepted by the hospital-faculty ethics committee of the CHU de Liège (Approval number 2019/223). Patients with a CD diagnosis and a first surgical resection between 1992 and 2018 were screened for inclusion. We decided to divide them into three cohorts which correspond to periods characterized by different clinical management: between 1992 and 2001 (first cohort), between 2002 and 2011 (second cohort), and between 2012 and 2018 (third cohort). The period 1992–2001 corresponds to the period when there was no systematic early colonoscopy after surgical resection. The second period (2002–2011) corresponds to the period when a majority of patients had an early post-surgery colonoscopy but just a minority benefited from post-operative use of biologic therapies (mainly anti-TNF). Finally, the third period (2012–2018) corresponds to the period when not only an early post-surgery colonoscopy was done more systematically and but also the patients benefited from an extended use of biologics during the post-operative period. The exclusion criteria were: (1) follow-up of less than six months or follow-up performed externally, (2) an ileo- or colostomy, (3) an ileocolic anastomosis not visible by endoscopy, and (4) an age of less than 18 years at the time of surgery.

### 2.2. Data Collection

Data were collected from January 1992 to August 2020. About 30 variables appearing to be relevant for postoperative recurrence were extracted from the medical notes of the selected patients. The variables studied were: sex, active smoking at the time of diagnosis, age at diagnosis and at the time of the first surgery, family history of CD, duration of CD before the first surgery, presence of extra-intestinal manifestations, appendectomy before the first surgery, the Montreal classification at diagnosis (location, behavior, perianal lesions), the anatomical site resected at the first surgery and its length, the surgical technique, the type of anastomosis (side-to-side, side-to-end, end-to-end), the treatment received in the context of CD before surgery, the treatment received for primary prevention after surgery, the Rutgeerts score at post-operative colonoscopy when performed and the treatment started after it. When the anastomosis was colonic-colonic or colonorectal, we used the adapted colonic Rutgeerts score.

### 2.3. Outcome Measure

The studied outcome was surgical recurrence defined as the reappearance of symptoms refractory to medical treatment leading to another intestinal resection with surgical and pathological confirmation of relapse of CD. We included all surgical recurrences and not only those, most frequently occurring at the site of the first anastomosis.

### 2.4. Statistics Analysis

Patient characteristics were described as mean and standard deviation or median and IQR for continuous variables and frequency (%) for categorical variables. The time to surgical recurrence was compared between the three cohorts by the Log-Rank test and was displayed on a Kaplan–Meier curve. The factors associated with surgical recurrence were analyzed by the univariate and multivariate Cox regression model. The results were considered significant at the critical rate of 5% (*p* < 0.05). Calculations were performed using SAS Version 9.4 (SAS Institute, Cary, NC, USA) for statistics and R Version 3.6 for graphics.

## 3. Results

### 3.1. Patients

**Figure 1:**
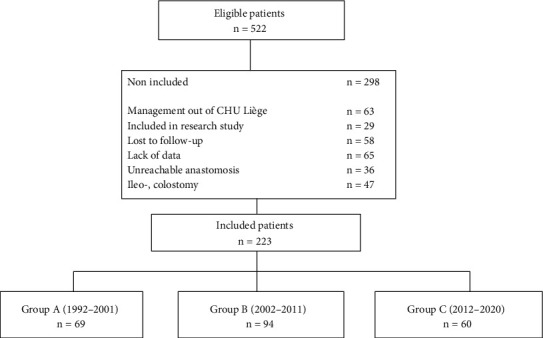
Patients flow chart.

From January 1992 and September 2018, 521 patients with CD underwent their first intestinal resection at our university hospital, CHU of Liège. Of these, 298 could not be selected due to lack of data in the chart, lost to follow up, a revised diagnosis, participation in a study modifying usual follow-up, and management out of our university hospital. We could include 223 patients according to the criteria of the study. The patients' flowchart is shown in Figure 1.

They were 69 in the period 1992–2001 (group A), 94 in the second period from 2002 to 2011 (group B), and 60 in the last period from 2012 to 2020 (group C). The entire sample includes 141 women (63.2%) for 82 men (36.6%). The mean age at the time of surgery was 35 years (±12). All the characteristics of the patients studied in the three cohorts are shown in Table 1. Eight patients died during the study period.

### 3.2. Surgical Relapse

**Figure 2:**
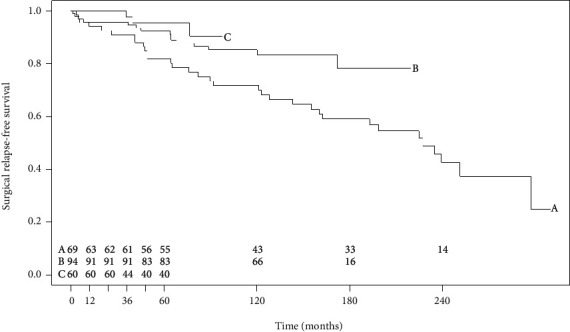
Kaplan–Meier curve of surgical relapse observed in the three cohorts. There was a statistically significant difference between group C and the other two cohorts (Log-Rank test: *p* = 0.0089).

The median survival time without surgical recurrence for the entire cohort was 251 months (IQR 160–251). In the 1992–2001 group, 33 of 69 patients (47.8%) had to undergo a second resection compared to 15 of 94 patients (16.0%) in the second cohort and 3 of 60 patients (5.0%) in the 2012–2020 cohort. As illustrated in Figure 2, a statistically significant increase in the time-to-surgical relapse was observed in group C compared to the other two cohorts (Log-Rank test: *p* = 0.0089). The one-year surgical recurrence rates were 5.9%, 4.3%, and 0% in groups A, B, and C, respectively. Three-year surgical recurrence rates were 9.0%, 4.3%, and 2.3% in groups A, B, and C, respectively. The five-year surgical recurrence rates were 18.2%, 7.6%, and 4.7% in groups A, B, and C, respectively.

A post-operative ileocolonoscopy was performed in 127 patients with a Rutgeerts score (or colonic adapted Rutgeerts score) in 126 of them. Ten patients (8.0%) had a reoperation.

The factors associated with the risk of surgical recurrence in univariate and multivariate are shown in Tables 2, 3, and 4.

## 4. Discussion

Our study shows a significant decrease in surgical recurrence in CD over the last 30 years, in 3 successive cohorts. Over the same period of time a change in clinical practice occurred at our centre, as recommended by ECCO guidelines, with the generalisation of the post-operative colonoscopy monitoring and the adaptation of post-operative preventive treatment. Our study also shows a significant decrease in the time-dependent surgical recurrence when a post-operative colonoscopy was performed, which was more frequent as of the year 2000.

Surgical recurrence in CD represents a very relevant outcome, since it is associated with disease progression and accumulation of tissue damage [13]. It also probably represents a more robust judgement criterion than clinical recurrence in a retrospective study. The surgical recurrence rates found in the literature vary between 11 and 32% at five years [14]. A recent UK, nation-wide study performed between 2007 and 2016 revealed a 9% surgical recurrence rate at 5 years [15]. Our rates are lower, particularly in the two most recent periods (18% in group A, 7.6% in group B, and 4.7% in group C). As we deliberately choose to compare three different cohorts corresponding to major changes in our practice of post-operative management over time, this may reflect the impact of the implementation of post-operative colonoscopy and subsequent treatment adaptation. Indeed, in the UK nation-wide study performed in the period between our last two periods, a post-operative colonoscopy was only performed in 22% of cases while it was 69% and 85% in our last two cohorts, respectively. Our second cohort started after the year 2001, a time when we started to implement more systematically early post-operative colonoscopy.

The impact of performing a colonoscopy has already been suggested on clinical relapse [11] and endoscopic relapse [12]. Although the use of early postoperative endoscopy is recommended in the guidelines of several IBD scientific societies because of the predictive value of endoscopic recurrence on subsequent clinical relapse [7, 10, 24], there is little data in the literature on its use and its impact on the clinical outcome of patients. A recent Cochrane systematic review and meta-analysis on the impact of colonoscopy-guided post-operative treatment suggested a trend toward a lower clinical recurrence and toward a lower endoscopic activity score at 18 months [16]. However, the main conclusion was that there was insufficient data and that particularly the impact on surgical recurrence had not been appropriately studied. To our knowledge, our study is one of the first to show the positive impact on surgical relapse, after performing a colonoscopy between six months and one year after surgery.

Obviously, colonoscopy cannot have any impact if the therapy is not subsequently appropriately adapted. A retrospective study dating from 2012, one of the first of its kind, studied the impact of early colonoscopy and the adjustment of treatments according to it [25]. In this study, colonoscopy had no impact on clinical relapse, but this study also highlighted the lack of standardized treatments based on the lesions discovered by endoscopy and the inadequate intensification of therapy. In fact, this therapeutic escalation mainly consisted of taking immunosuppressants and did not include anti-TNF therapy.

In our practice, during the 2000s, the main post-endoscopic treatment for significant endoscopic recurrence was azathioprine (at a minimal dose of 2 mg/kg, unless not tolerated) and mesalazine for minor recurrences. These treatments do not appear to have had an impact on the surgical recurrence rate. This confirms data in the literature showing a marginal effect for mesalazine [8, 26] to a slight effect for azathioprine [8, 19]. As for anti-TNFs, some studies showed efficacy in preventing endoscopic recurrence but data are weaker for clinical relapse. The first study by Regueiro et al. evaluated the effect of infliximab on clinical and early endoscopic relapse in 24 post-operative patients. Endoscopic relapse at one year was significantly lower in the infliximab group than in the placebo group, while clinical relapse at one year, albeit numerically lower in the Infliximab group, was not significantly different [28]. This was followed by the Prevent study which showed a significant decrease in endoscopic recurrence but not in clinical recurrence which was the primary end point [29]. Beyond this, infliximab has been shown to be more effective than mesalazine and azathioprine once endoscopic relapse at six months is proven [31]. The POCER study demonstrated superior efficacy on endoscopic relapse of adalimumab compared to thiopurines in patients at high risk of relapse (smoking, perforating phenotype, history of previous resections) [27]. This conclusion was, however, not fully confirmed by a GETTECU trial [31].

The more pronounced decrease in the surgical recurrence rate in the most recent cohort as compared to the intermediate cohort (the five-year surgical recurrence rates were 7.6% and 4.7% in groups B and C, respectively) may be linked not only to further increasing number of post-operative colonoscopies but also to a more frequent use of anti-TNF after colonoscopy (10.8% and 35%, respectively). Indeed, a few years after the first open-label study showing a striking decrease in post-operative endoscopic recurrence with the use of anti-TNF [28], as of 2012, we started to use these drugs more systematically in the post-operative periods. Currently, most algorithms implement prophylactic therapy in patients at high risk of relapse with “step-up” therapy in all patients if they have a recurrence seen by colonoscopy [14, 33]. Anti-TNF drugs alone or in combination have been shown to be associated with a decrease in endoscopic recurrence [34] which is predictive of symptomatic clinical relapse and the need for future resection [8, 36].

Besides the impact of performing a post-operative ileocolonoscopy, we also showed that the rate of surgical recurrence was increased with a higher Rutgeerts score at the post-operative endoscopy. The impact of this score was initially demonstrated in the Rutgeerts original study [10]. The endoscopic assessment score was the main significant factor associated with clinical relapse. In addition, the importance of the endoscopic score was subsequently confirmed by a second study a few years later [7]. This factor therefore still currently represents the best predictor of postoperative recurrence. The treatment optimisation after endoscopy may explain why we did not observe any significant impact of the immediate post-operative treatment by thiopurines or biologics on further surgical recurrence, while it has a proven impact on the endoscopic recurrence [33, 34].

Other factors influencing recurrence after a first surgery have been mainly studied for endoscopic and clinical recurrence, but much less for surgical recurrence. Factors favouring early post-surgical relapse in CD are well known and include smoking, a penetrating phenotype at diagnosis, a history of digestive resection (which may include appendectomy), extensive resection, and associated perianal lesions [14]. Like our study, a large number of other studies identified smoking as an independent risk factor for endoscopic, clinical, and surgical recurrence [18, 19]. A meta-analysis by Reese et al. bringing together 2962 patients has shown that smokers increased their risk of surgical recurrence by 2.5 times at 10 years, and by twice the risk of post-operative clinical relapse compared to non-smokers [20]. In our study, in the whole cohort, the time-dependent risk of surgical recurrence was also associated with treatment with thiopurine before the first surgical resection. This may reflect a more severe disease course. In 2017, a retrospective study showed that previous exposure before intestinal surgery to two or more anti-TNF*α* in CD patients was associated with a higher risk of postoperative clinical recurrence [21].

In our data, an increased risk of surgical recurrence was also observed with the intake of corticosteroids and methotrexate after colonoscopy. Studies describing the impact of corticosteroids given after surgery or following colonoscopy have shown no impact on endoscopic relapse or clinical relapse [22]. In the randomized, double-blind trial by Heller et al., the rate of endoscopic recurrence at 3 and 12 months was compared in 129 patients treated with Budesonide 6 mg/day vs placebo. There was no significant difference in the rate of endoscopic recurrence at 3 and 6 months [23]. The impact of Methotrexate on surgical recurrence has been little studied. They represented only a small minority of our patients. There are several hypotheses to explain why these treatments emerged as associated with an increased risk of surgical recurrence: it may reflect the endogenous aggressiveness of CD, suggest that these drugs are inferior to others (like purines or anti-TNF) in preventing recurrence, or suggest that these drugs could persistently modify the patient's immune status and influence the relapse.

Our study has some limitations. It was retrospective and the decisions to treat post-operatively, perform an early colonoscopy, and give treatment after colonoscopy, which were not standardized in a protocol. The final population included is less than a half of those originally operated, and in this context, a selection bias is always possible. In fact, patients who could not be included because they were lost to follow up or had their follow-up in other hospitals may have had a more benign disease course. This could have led to an overestimation of the relapse rate. Nevertheless, our relapse rate was close to those previously described in the literature, and this impact should have been the same in the three successive cohorts, whose comparison was our main aim.

In conclusion, we have observed a significant decrease in surgical recurrence after the first surgical resection for CD over the last 30 years in our routine practice, and this has been associated with the implementation of an early ileocolonoscopy between 6 and 12 months after surgery. Together with this early endoscopy, another key element to impact the outcome might be the choice of the treatment prescribed after endoscopy. Controlled trials are needed, to compare these treatments not only in preventing endoscopic recurrence but also in clinical and surgical recurrence.

## Figures and Tables

**Table 1:** Baseline characteristics of the patients. (CC = corticosteroids; MTX = méthotrexate; IQR: interquartile range).

Baseline characteristics	Group A, *n* = 69	Group B, *n* = 94	Group C, *n* = 60	*p*-Value
Male, *n* (%)	23 (33.3)	35 (37.2)	24 (40.0)	0.73
Age at diagnostic, years, Median (IQR)	30 (20–34)	28 (20–33)	28 (20–33)	0.82
Age at first surgery, years, median (IQR)	35 (26–42)	34 (25–42)	36 (27–43)	0.77
Duration of disease before surgery, years, Median (IQR)	5 (0–8)	6 (0–9)	8 (1–11)	0.17
Death (%)	5 (7.2)	2 (2.1)	1 (1.7)	0.14
Family history of IBD, *n* (%)	18 (30.0)	27 (37.5)	17 (30.4)	0.58
Smokers, *n* (%)	37 (61.7)	50 (53.8)	30 (50.0)	0.42
Extraintestinal manifestations, *n* (%)	17 (27.9)	23 (25.6)	12 (20.0)	0.58
Appendicectomy before surgery, *n* (%)	27 (40.3)	23 (24.5)	7 (11.7)	**0.0011**
Disease location (Montreal classification), *n* (%)	69	94	60	0.099
				
L1 = small bowel	36 (52.2)	59 (62.8)	36 (60.0)	
				
L2 = colon	4 (5.8)	7 (7.4)	2 (3.3)	
				
L3 = small bowel + colon	27 (39.1)	25 (26.6)	17 (28.3)	
				
L4 = upper gastrointestinal tract	2 (2.9)	2 (2.1)	0 (0.0)	
				
L1 + L4	0 (0.0)	0 (0.0)	2 (3.3)	
				
L3 + L4	0 (0.0)	1 (1.1)	3 (5.0)	
				
Behaviour (Montreal classification), *n* (%)	61	91	52	0.48
				
B1 = non-stenosing non-penetrating	30 (49.2)	50 (54.9)	31 (59.6)	
				
B2 = Stenosing	17 (27.9)	19 (20.9)	14 (26.9)	
				
B3 = penetrating	14 (23.0)	20 (22.0)	7 (13.5)	
				
B2 + B3	0 (0.0)	2 (2.2)	0 (0.0)	
Perianal disease, *n* (%)	14 (20.3)	19 (20.4)	5 (8.3)	0.11
Treatment before surgery, *n* (%)				
				
Aminosalicylates	58 (84.1)	80 (85.1)	40 (66.7)	**0.012**
				
Corticosteroids	52 (75.4)	65 (69.1)	52 (86.7)	**0.047**
				
Methotrexate	1 (1.4)	10 (10.6)	18 (30.0)	**<0.001**
				
Thiopurines	8 (11.6)	37 (39.4)	34 (56.7)	**<0.001**
				
Anti-TNF	0 (0,0)	19 (20.2)	39 (65.0)	**<0.001**
				
Vedolizumab	0 (0.0)	0 (0.0)	2 (3.3)	0.064
				
Antibiotics	30 (43.5)	40 (42.6)	41 (68.3)	**0.0035**
Location resection	69	94	60	**0.0001**
				
Small bowel	5 (7.2)	8 (8.5)	1 (1.7)	
				
Colon	6 (8.7)	4 (4.3)	3 (5.0)	
				
Ileocecal	26 (37.7)	71 (75.5)	39 (65.0)	
				
Ileocolic	31 (44.9)	10 (10.6)	15 (25.0)	
				
Colon + upper intestinal tract	0 (0.0)	1 (1.1)	0 (0.0)	
				
Colon + rectum resection	0 (0.0)	0 (0.0)	1 (1.7)	
				
Ileocecal + colon + rectum resection	1 (1.4)	0 (0.0)	0 (0.0)	
				
Ileocolic + rectum resection	0 (0.0)	0 (0.0)	1 (1.7)	
Length of resection cm	34.73 ± 16.856	31.93 ± 19.747	29.05 ± 14.580	0.20
Surgical technics				**<0.001**
				
Laparotomy	51 (82.3)	38 (42.2)	19 (31.7)	
				
Laparoscopy	11 (17.7)	52 (57.8)	41 (68.3)	
Surgical technic of anastomosis	46	77	57	**<0.001**
				
Handsewn	44 (95.7)	61 (79.2)	11 (19.3)	
				
Stapled	2 (4.3)	16 (20.8)	46 (80.7)	
Type of anastomosis	46	77	54	**<0.001**
				
Side-to-end	21 (45.7)	33 (42.9)	2 (3.7)	
				
Side-to-side	1 (2.2)	25 (32.5)	49 (90.7)	
				
End-to-end	24 (52.2)	19 (24.7)	3 (5.6)	
Treatment after post surgery *n* (%)				
				
Aminosalicylates	45 (65.2)	12 (12.8)	4 (6.7)	**<0.001**
				
Corticosteroids	8 (11.6)	8 (8.5)	2 (3.3)	0.22
				
Methotrexate	1 (1.4)	1 (1.1)	3 (5.0)	0.24
				
Thiopurines	3 (4.3)	13 (13.8)	10 (16.7)	0,065
				
Anti-TNF	0 (0,0)	5 (5.3)	18 (30.0)	<0.001
				
Vedolizumab	0 (0.0)	0 (0.0)	2 (3.3)	0.064
				
Antibiotics	5 (7,2)	10 (10.6)	1 (1.7)	0.11
Time between surgery and colonoscopy (months)	6.42 ± 3.045	8.00 ± 3.962	7.56 ± 3.195	0.38
Colonoscopy 6 to 12 months after surgery, *n* (%)	11 (15.9)	65 (69.1)	51 (85.0)	**<0.001**
Rutgeerts score at colonoscopy after surgery				0.16
				
Stage *i*,0	0 (0.0)	16 (25.0)	9 (17.7)	
				
Stage *i*,1	2 (18.2)	14 (21.9)	10 (19.6)	
				
Stage *i*,2	2 (18.2)	16 (25.0)	15 (29.4)	
				
Stage *i*,3	4 (36.4)	13 (20.3)	6 (11.8)	
				
Stage *i*,4	3 (27.3)	5 (7.8)	11 (21.6)	
Treatment following results of colonoscopy	11	65	51	**0.0007**
				
Yes	11 (100.0)	65 (100.0)	42 (82.4)	
				
No	0 (0.0)	0 (0.0)	9 (17.6)	
Treatment started after results of colonoscopy				
				
Aminosalicylates	4 (36.4)	15 (23.1)	0 (0.0)	**0,0003**
				
Corticosteroids	6 (54.6)	3 (4.6)	3 (5.9)	**<0.0001**
				
Methotrexate	0 (0.0)	7 (10.8)	3 (5.9)	0.37
				
Thiopurines	2 (18.2)	8 (12.3)	2 (3.9)	0.18
				
Anti-TNF	0 (0.0)	7 (10.8)	18 (35.3)	**0.0010**
				
Vedolizumab	0 (0.0)	0 (0.0)	1 (2.0)	0.47
				
Ustekinumab	0 (0.0)	0 (0.0)	0 (0.0)	0.22

The median time of follow-up after surgery was 235 months (IQR 166–265) in cohort A, 131 months (IQR 98–163) in cohort B, and 59 months (IQR 31–81) in cohort C.

**Table 2:** Predictive factors of post-operative surgical recurrence in univariate analysis.

	*N*	Comparison	*P*-Value	Hazard ratio	95% CI
Group	223	1 vs 3	0.0124	3.76	1.11–12.732
		2 vs 3		1.683	0.48–5.902
		1 vs 2		2.234	1.177–4.238
Smoking	213	1	0.0204	2.14	1.125–4.07
Resection of small bowel	223	1	0.0499	2.354	1–5.538
Laparotomy	212	1	0.0288	2.109	1.08–4.118
Colonoscopy 6 to 12 months after surgery	223	1	0.0002	0.272	0.137–0.538
Score de Rutgeerts	126	1	0.0035	2.554	1.362–4.787
Steroid treatment started after ileocolonoscopy	127	1	0.0005	8.992	2.594–31.176
Methotrexate treatment started after ileocolonoscopy	127	1	0.031	4.335	10.144–16.431

**Table 3:** Predictive factors of postoperative surgical recurrence multivariate analysis in the entire cohort.

	Hazard ratio	95% CI	*p*-Value
Smoking	2.250	1.159–4.367	0.0166
Thiopurine treatment before surgery:	2.637	1.282–5.424	0.0084
Colonoscopy 6 to 12 months after surgery	0.311	0.138–0.701	0.0049

**Table 4:** Predictive factors of postoperative surgical recurrence multivariate analysis in patients with post-operative ileocolonoscopy.

Variable	Hazard ratio	95% CI	*p*-Value
Steroid treatment started after ileocolonoscopy	9.992	1.728–57.764	0.0101
Methotrexate treatment started after ileocolonoscopy	11.929	1.728–57.764	0.0030
Rutgeerts score	2.362	1.203–4.639	0.0125

## Data Availability

The data used to support the findings of this study are included within the article.
